# The impact of health volunteering of radiology students on improving their self-skills and practical capabilities in the Kingdom of Saudi Arabia

**DOI:** 10.3389/fmed.2023.1243014

**Published:** 2024-02-29

**Authors:** Amel F. Alzain, Nagwan Elhussein, Zuhal Y. Hamd, Ibtisam Abdallah Fadulelmulla, Awatif M. Omer, Ahoud Alotaibi, Amani Alsuhaymi, Maram Aljohany, Najwa Alharbi, Amna Mohamed Ahmed, Rehab Hussien, Badria Awad Elamin, Afaf Mohamed Ahmed Medani, Mayeen Uddin Khandaker

**Affiliations:** ^1^Department of Diagnostic Radiology Technology, College of Applied Medical Sciences, Taibah University, Al-Madinah Al-Munawwrah, Saudi Arabia; ^2^Department of Diagnostic Radiology, College of Applied Medical Sciences, University of Ha’il, Ha’il, Saudi Arabia; ^3^Department of Radiological Sciences, College of Health and Rehabilitation Sciences, Princess Nourah Bint Abdulrahman University, Riyadh, Saudi Arabia; ^4^Department of Radiological Sciences, College of Applied Medical Sciences, King Khalid University, Abha, Saudi Arabia; ^5^Applied Physics and Radiation Technologies Group, CCDCU, School of Engineering and Technology, Sunway University, Bandar Sunway, Selangor, Malaysia; ^6^Faculty of Graduate Studies, Daffodil International University, Dhaka, Bangladesh

**Keywords:** health volunteering, radiology students, self-skills, practical capacities, theoretical knowledge

## Abstract

**Background:**

Volunteering is a beneficial activity with a wide range of positive outcomes, from the individual to the communal level. In many ways, volunteering has a positive impact on the development of a volunteer’s personality and experience. This study aimed to evaluate the impact of health volunteering on improving the self-skills and practical capacities of students in the western region of the Kingdom of Saudi Arabia.

**Materials and methods:**

The study was a descriptive cross-sectional electronic web-based survey that was submitted on a web-based questionnaire; 183 students answered the survey, and then, the data were analyzed using SPSS.

**Results:**

This study shows that 95.6% of participants agree and strongly agree that the health volunteering experience was useful, 2.7% of the participants neither agree nor disagree, and 1.6% disagree and strongly disagree. Regarding the distribution of the participants on skills learned from volunteering experience, the largest proportion of student (36.1%) volunteers in the health sector acquired communication skills and the smallest proportion of student (14.8%) volunteers in the acquired time management skills. Regarding the disadvantages, 81.4% of the participants do not think there were any disadvantages to their previous health volunteering experience, while only 18.6% of them think there were any disadvantages to their previous health volunteering experience. Additionally, the study found that the type of the sector affects the skills acquired from health volunteering.

**Conclusion:**

Research revealed that the majority considered volunteering a great experience. Volunteering increased the self-skills and practical capacities of radiology students, which proved the hypothesis.

## 1 Introduction

Philosophers and researchers have discussed the ideas that are making people feel happy and comfortable since ancient times and volunteerism is one among them. Volunteering is an activity where the volunteers spend their time free of cost to help another individual, organization, or cause. It is one of the beneficial activities that have a wide range of positive results, from a personal to a community level (1, 2). A volunteer is an individual who is socially aware, energetic, and enthusiastic about the world in general, as well as willing to make sacrifices for the good of others (3). In several regions of the world, student volunteerism as a method of community interaction is not only a crucial component of higher education’s goal but also a requirement for graduation that plays a crucial role in experiential learning (3, 4). Volunteering is crucial for students and helps to develop their personalities, communication skills, and capacities because education alone cannot meet all of their needs to be successful and healthy students (5). Several categories of organizations such as welfare and community, sports, recreation, education, and training, all rely on volunteers. Although volunteering is meant to assist others, however, it also brings benefits to the volunteers and has recently been the subject of significant discussion (6, 7). Professional development, skill and knowledge growth, personality and fulfillment, and academic achievement are among the areas of influence, according to the literature (8). Students who volunteer are more capable, employable, and efficient in completing their academic objectives (3). The possibility to become competent and employable can be taken advantage of after finishing all the learning objectives (9, 10). The phrase “student volunteering” has been used to describe a variety of endeavors, including work with clubs and organizations and university service (ranging from environmental groups to photography, business, and sports centers) (6, 11). The health of a community and the continuation of civilization require volunteerism.

A young person’s personality, character, identity, values, and drive can all be positively impacted by volunteering (12). It also gives them new opportunities to practice their communication and enhance their problem-solving and cooperation skills (13). The benefits of helping others are numerous and include improved personal wellbeing, decreased mortality, increased physical function, higher levels of self-rated health, less depressive symptoms, and higher levels of life satisfaction (14, 15). Volunteering can also offer a chance to hone job search strategies, as the process of seeking, securing, and applying for volunteer positions closely mirrors that of job hunting (16). According to a study, 59% of medical students at the National University of Ireland volunteer during pandemics (17). Medical students have demonstrated a strong drive to volunteer for medical-related causes. The possibility to interact with individuals from many cultures, a natural desire to give back, educational possibilities, the chance to advance one’s clinical abilities (18), and the chance to collaborate with numerous healthcare professionals are among the main drivers (19).

On the other hand, medical students could encounter several obstacles that hinder them from taking part in volunteer work. Undoubtedly, psychological considerations are the most frequent of these obstacles (20). Most researchers around the world focus on the effect of volunteering on healthcare students, especially medical and nursing students (21). However, health volunteering has been recognized as an effective means of providing practical experience and enhancing the personal and professional development of students in various healthcare fields; there is no such research discussing the effects of volunteerism on the behavior, skills, and personal development of radiology students. In addition to this, no research has focused on the impact of health volunteering specifically on diagnostic radiology students in the western region of Saudi Arabia. This study may provide a rationale by examining existing evidence on the benefits of health volunteering for students in diagnostic radiology while highlighting the added value of conducting this research in the specified region. Therefore, the current study was designed to evaluate the effects of (i) health volunteering on improving the self-skills of students, (ii) health volunteering experience on practical abilities, and (iii) to demonstrate the advantages and disadvantages of health volunteering based on student perception.

This study holds several added values for the field of diagnostic radiology education and healthcare volunteering in the western region of Saudi Arabia. First, by specifically focusing on this region, the study will provide localized evidence on the impact of health volunteering on diagnostic radiology students. This will enable educational institutions and healthcare organizations in the region to tailor their volunteering programs focusing on the specific needs and contexts of the students and communities. Additionally, the study will contribute to the existing body of knowledge by highlighting the unique experiences and challenges faced by diagnostic radiology students in the western region of Saudi Arabia, thereby expanding the prevailing understanding of the impact of health volunteering in diverse cultural and healthcare contexts.

## 2 Methodology

### 2.1 Study design and population

The study was conducted in the western region of the Kingdom of Saudi Arabia because it attracts a diverse population, including residents, pilgrims, and tourists. By focusing on the impact of health volunteering in this region, the study can shed light on the specific skills and competencies that students need to develop in order to effectively serve this diverse population.

The study was a descriptive cross-sectional electronic web-based survey. A web-based questionnaire was prepared (see Supplementary File) that dealt with the impact of health volunteering on improving the self-skills and practical capacities of students in the western region of the Kingdom of Saudi Arabia. The research study was conducted from February to May 2022. The questionnaire was distributed across the western region of Saudi Arabia through digital tools. All the radiology students were included in the study who have been involved in health volunteering anywhere in the western region of Saudi Arabia regardless of gender, educational level, and type of sector. The study sample size is 300, with 95 and 5% margin of error.

### 2.2 Hypothesis

Health volunteering may improve the self-skills and practical capacities of the students.

### 2.3 Data collection process

The data were collected using a data collection sheet (questionnaire) designed specifically for the study and submitted via the web for participant inclusion via an electronic survey (Google Forms). The questionnaire was accompanied by a cover letter outlining the objectives of the study, noting that participation was entirely optional, and providing the authors’ contact details. If participants had any questions about the questionnaire, they were urged to get in touch with the researchers. The Google Forms questionnaire was disseminated through social media (Telegram, WhatsApp, Facebook, email, and Twitter) across the western region of Saudi Arabia as an electronic link. The participants often answered the survey by entering the response directly.

### 2.4 Ethical compilation

The Scientific Research Ethics Committee, reference number 2021/127/314/DRD, approved the research proposal. Informed consent was obtained from all participants prior to their voluntary participation in the study. The study does not contain any information that might be used to identify the participants.

### 2.5 Statistical analysis

Statistical analysis was carried out using SPPSS ver. 26.7, where a test of significance is applied through the use of ANOVA, a one-way analysis of variance. It is a statistical technique that divides the observed variance data into various components for use in further testing. ANOVA is used to find out how the dependent and independent variables are related when there exist three or more data groups within the study.

## 3 Results

Volunteering is one of the beneficial activities that have a wide range of positive results from a personal to a community level. A volunteer is someone who is socially aware, energetic, and enthusiastic about the world in general, as well as willing to make sacrifices for the good of others. In several regions of the world, student volunteerism as a method of community interaction is not only a crucial component of higher education’s goal but also a requirement for graduation that plays a crucial role in experiential learning. A Google Forms questionnaire was sent to the radiology students. The questionnaire was answered by 183 students. Of 183 participants, 106 (57.59%) were female participants, while 77 (42.1%) were male participants (Table 1).

**Table 1:** The demographic characteristics of the study participants.

Variables	Types	Frequency	Percentage
Gender	Female	106	57.9
	Male	77	42.1
	Total	183	100
Location	Almadinah Almunawara	87	47.5
	Jeddah	66	36.1
	Taif	13	7.1
	Makkah Al-Mukarramah	11	6.0
	Qonfotha	6	3.3
	Total	183	100
Sector	Private Healthcare	66	36.1
	Ministry of Health Healthcare	105	57.4
	Governmental Healthcare	67	36.6
	Total	238	130
Frequency of time	One time	64	35.0
	Two times	46	25.1
	Three times	45	24.6
	Three times+	28	15.3
	Total	183	100
Scope of volunteering	Within and outside of your specialty	70	38.3
	Within specialty	67	36.6
	Outside specialty	46	25.1
	Total	183	100

Moreover, 47.5% of the participants were from Almadinah Almunawara, followed by 36.1% from Jeddah, 7.1% from Taif, 6% from Makkah Al-Mukarramah, and 3.3% from Qonfotha. Similarly, the maximum number of participants, approximately 57.4%, volunteered for the Ministry of Healthcare Facility, followed by 36.6% for the government healthcare facilities (military and education), and 36.1% (the lowest) volunteered for private healthcare facilities. Furthermore, in the distribution of the participants on the frequency of volunteering time in the health sector, 35% of the participants have volunteered in the health sector only once, 25.1% of them have volunteered two times, 24.6% volunteered three times, and 15.3% volunteered more than three times (Table 2). In addition, 36.6% of the participants determined that it was within their specialty, followed by 38.3% who determined that it was both within and outside their specialty, and 25.1% of them determined that it was outside their main specialty.

**Table 2:** The usefulness of health volunteering for the study participants.

Question	Total frequency	Total percent
Strongly agree	110	60.1%
Agree	65	35.5%
Neither agree nor disagree	5	2.7%
Disagree	2	1.1%
Strongly disagree	1	0.5%
Total	183	100%

In regards to the usefulness of health volunteering experiences, majority of the study participant 60.1% strangely agree and 35.5% agree that it was useful, while only 2.7% and 1.1% neither agree nor disagree and disagree respectively.

Table 3 demonstrates that 56% of them stated that they have gained practical skills, 24% of them stated that they have gained new skills, 13.7% of the respondents stated that they have gained theoretical knowledge, only 4% of them stated that they have gained many friends, and 2.3% of them stated other reasons, such as Table 4 illustrates that the primary justification for individuals who responded with “disagree” or “strongly disagree” was twofold: the lack of practical skills acquired and the absence of theoretical knowledge gained. Concerning the main skills that gained from health volunteering, the results shows that communication is selected by 36.1% of the study participants, problem solving selected by 29 %, then the leadership and time management selected by 20.2% and 14.8% of the study participants respectively.

**Table 3:** The main reasons if the answer was “agree” or “strongly agree.”

Questions		Strongly agree		Agree		Frequency	Percentage
		Frequency	Percent	Frequency	Percent		
Gained practical skills		65	37.14%	33	18.86%	98	56.00%
Gained new skills		25	14.29%	17	9.71%	42	24.00%
Gained theoretical knowledge		13	7.43%	11	6.29%	24	13.71%
Gained many friends		6	3.43%	1	0.57%	7	4.00%
Others	No experience	0	0.00%	2	1.14%	2	1.14%
	All answers were yes	0	0.00%	1	0.57%	1	0.57%
	Counted volunteering hours	1	0.57%		0.00%	1	0.57%
Others		1	0.57%	3	1.71%	4	2.29%
Total		110	63%	65	37%	175	100%

**Table 4:** The main reasons, if the answer was “disagree” or “strongly disagree.

Disagree or strongly disagree	Disagree		Strongly disagree		Frequency	Percentage
	Frequency	Percent	Frequency	Percent		
Did not gain practical skills	1	33.33%	0	0.00%	1	33.33%
Did not gain theoretical knowledge	1	33.33%	0	0.00%	1	33.33%
Total	2	66.7%	1	33.3%	3	100%

Table 5 demonstrates that there is no relationship among the extent of benefit from the volunteering experience, gender (*p* = 0.478), academic year (*p* = 0.759), volunteer experience location (*p* = 0.606), number of times volunteered in the health sector (*p* = 0.077), experience in health volunteering (*p* = 0.229), number of healthcare facilities volunteered for (*p* = 0.115), longer duration of healthy volunteering experience (*p* = 0.220), and type of sector (*p* = 0.338).

**Table 5:** The relationship between demographic data and the usefulness of the volunteering experience.

Health volunteering experience was useful						
Question	Health volunteering experience was useful					*P*-value
	Strongly disagree	Disagree	Neither agree nor disagree	Agree	Strongly agree	
**Gender**						
Male	0	2	2	27	46	0.478
Female	1	0	3	38	64	
**Academic year**						
Fourth year	1	1	1	26	54	0.759
Intern year	0	1	1	18	21	
Second year	0	0	1	9	9	
Third year	0	0	2	12	26	
**Volunteer experience location**						
Almadinah Almunawara	0	1	2	35	49	0.606
Jeddah	1	0	3	20	42	
Taif	0	0	0	3	10	
Makkah Al-Mukarramah	0	1	0	4	6	
Qonfotha	0	0	0	3	3	
**How many times have you volunteered in the health sector?**						
One time	0	1	3	25	35	0.077
Two times	0	0	1	22	23	
Three times	0	1	0	8	36	
More than three times	1	0	1	10	16	
**Scope of your experience in health volunteering**						
Both	0	0	2	19	49	0.229
Within specialty	0	1	2	31	33	
Outside specialty.	1	1	1	15	28	
**Number of healthcare facilities have you volunteered for**						
1	0	0	3	24	35	0.115
2	0	1	1	27	33	
3	0	0	0	9	26	
More than 3	1	1	1	5	16	
**Duration of your longest health volunteering experience**						
2 weeks—1 month	1	1	4	39	42	0.220
1—2 months	0	1	1	16	36	
2—3 months	0	0	0	8	31	
More than 3 months	0	0	0	2	1	
**What sector did you volunteer for?**						
Private Healthcare Facility	0	1	1	18	46	0.338
Ministry of Healthcare	0	1	3	28	43	
Governmental Healthcare Facility	1	0	1	19	21	

## 4 Discussion

There is no such research discussing the effects of volunteerism on the behavior, skills, and personal development of radiology students. The current study is designed to evaluate the effects of health volunteering on improving the self-skills and practical abilities of radiology students in the western region of the Kingdom of Saudi Arabia. Data collection through Google Forms is a reliable and convenient way of collecting data from students (22). As observed the study shows that volunteerism has a positive impact on and practical abilities of radiology students in the western region of the Kingdom of Saudi Arabia. In addition, 35% of the participants volunteered in the health sector one time only, 25.1% of the participants volunteered two times, 24.6% volunteered three times, and 15.3% volunteered more than three times. This study’s results, shows that 57.9% of respondents were female, this result was compatible with those of Shi et al. and Boni et al., who found that the majority of study participants were female (12, 23). Moral obligation, individual interest, social commitment, and prosocial drive all have an impact on health professional students’ desire to volunteer (6). In addition, 36.6% of the participants volunteered inside their main specialty, 25.1% volunteered outside their main specialty, and 38.3% determined that it was both within and outside their specialty. These results are in contrast with the studies conducted by Drexler et al. and Wymer et al., who reported that most medical students wanted to volunteer during pandemics (24, 25). Moreover, 13.1% of the participants volunteered for more than three healthcare facilities, 19.1% volunteered for three healthcare facilities, 33.9% of the participants volunteered in one healthcare facility, and 33.9% volunteered in two healthcare facilities. Researchers reported that because of their loving, giving, and compassionate character, women were more likely to volunteer, but for a shorter time than male participants (26, 27).

The majority of students said that participating in volunteer work had tangible advantages, such as increasing one’s sense of helping others directly, getting professional experience, and honing teamwork skills (28, 29). A large percentage of students agreed that health volunteering experience was useful and the reasons for those who agree were as follows: 56% of them stated that they have gained practical skills, and 24% of them stated that they have gained new skills; in addition, 13.7% of them stated that they have gained theoretical knowledge, only 4% of them stated that they have gained many friends. Moreover, 2.3% of participants emphasized other reasons for the importance of volunteering in healthcare, including the provision of relationships with others and the counting of volunteering hours in careers opportunity. These results are in line with earlier research (30–32). A minority of the study participants mention that that health volunteering was un-useful, with the primary reasons for that the heath volunteering not improve practical or theoretical skills for them. Additionally, approximately 90.2% of the participants advised a colleague to have their experience, and those who advised the colleagues to have their experience were those who gained many benefits from health volunteering and agreed that volunteering was a useful experience (*p* = 0.000).

This finding is consistent with earlier study showing the beneficail effects of health volunteering experinces on people’s attitude and views about volunteering (33). The participants who reported gaining substantial benefits from their own volunteering experiences were more inclined to recognize the value and usefulness of such experiences. Consequently, they actively encouraged their colleagues to engage in health volunteering, potentially creating a ripple effect within their professional networks. In addition, two separate studies conducted by Ali et al. and Siqueira et al. reported that “The volunteering activities during the COVID-19 pandemic developed key skills from RCSI’s medical curriculum, significantly fostered medical students’ resilience and guided their career choices. Major areas of development included communication, teamwork, compassion, and altruism, which are not easily developed through the formal curriculum. A further area that was highlighted was the importance of evidence-based health in a pandemic” (34, 35). Additionally, it has been proposed that clinical exposure has a significant role in helping students become resilient and that volunteering during a pandemic enhances students’ professional growth and helps them build a professional identity.

The strong association between personal benefits and the recommendation of health volunteering experiences to colleagues underscores the potential transformative effects of these experiences on individuals’ self-perceptions, practical capacities, and professional development. Through health volunteering, diagnostic radiology students have the opportunity to enhance their skills, gain practical experience, and expand their understanding of healthcare delivery in diverse settings (36). These experiences can foster personal growth, increase self-confidence, and strengthen their commitment to serving their communities.

There was a perception of a lack of enthusiasm, personal health difficulties, a lack of protocol and understanding, and transportation issues (21). Regarding the main benefits, approximately 31.7% of the participants stated that gaining knowledge and understanding of other ways of life was the main benefit they gained, 28.4% of them stated to advance their career by improving their skills, 20.8% of them stated that they gain all the benefits of health volunteering, 10.9% of them stated to meet new people and build a community, and, finally, 8.2% of them stated to boost their self-esteem. Additionally, the study found that the type of sector affects the skills acquired from health volunteering (*p* = 0.001). Those who volunteered in the Ministry of Health facilities gained communication skills more than those who volunteered in private and governmental facilities, while those who volunteered in the private facilities gained problem-solving, leadership, and time management more than others. Those who volunteered in the governmental healthcare facility gained communication skills more than those in the private sector. Furthermore, the study shows that the sector also affects the benefits the students gain from health volunteering (*p* = 0.008). Those who volunteered in private healthcare facilities say that they gained knowledge and understanding of other ways of life, boosted their self-esteem, met new people, and built a community more than others who volunteered in the government and Ministry of Health facilities while those who volunteered in the ministry of health sector advance their career by improving their skills and gain all the benefits of health volunteering more than those in the private and governmental sectors. Those in the governmental sector gain benefits more than those in the private sector. Regarding the disadvantages to the previous health volunteering experience, 81.4% of the participants do not think there were any disadvantages to their previous health volunteering experience, while only 18.6% of them think there were any disadvantages to their previous health volunteering experience and they mentioned many reasons.

Additionally, 94.5% of the participants agreed that health volunteering improves their confidence, while only 5.5% of them did not agree that health volunteering improves their confidence and they mentioned the following reasons: “confidence is something we get through practicing our specialty in real life” and “I am confident in myself before I joined the volunteer field.” These results are in line with the research conducted by Yeung et al., Proulx et al., and Lee (37–39), who documented that volunteering improved self-confidence and happiness. Finally, the study showed that volunteering in general has many benefits on a personal level, including social interaction, increasing self-esteem, and improving relationships with others, and this is similar to the result (40). Another study showed that most of the students agreed that they had a useful health volunteering experience and that they developed their self-skills and learned new things. This is similar to the results of Chawłowska et al. (41) and Silva et al. (42).

## 5 Conclusion

This study has been conducted to evaluate the impact of health volunteering on improving the self-skills and practical capacities of radiology students in the western region of the Kingdom of Saudi Arabia. The results of the study proved the validity of the hypothesis. A large percentage of students agreed that the health volunteering experience was useful, as they improved their self-skills, and practical capacities and gained knowledge. There was no significant correlation between the participants response concerning the usefulness of health volunteering with gender, academic years, frequency of volunteering in health sector, scope of experince in health volunteering, duration of volunteering and type of sectors in which they performed health volunteering.

## 6 Limitations

Although the study used an online questionnaire, which was prepared for focused groups and made it possible to reach a sizable number of participants, the cohort was relatively small. However, it must be emphasized that using such a strategy prevents objectively validating the facts. As such, the obtained results may not be generalizable to other volunteering medical students’ groups.

## Data availability statement

The original contributions presented in the study are included in the article/Supplementary material, further inquiries can be directed to the corresponding author.

## Author contributions

AFA, ZH, NE, and AMA: conceptualization, resources, project administration, and funding acquisition. AFA, ZH, NE, IF, AO, AhA, AmA, MA, NA, RH, and BE: methodology. BE and AM: software. ZH: formal analysis. AFA, ZH, NE, IF, AO, AhA, AmA, MA, NA, RH, and BE: investigation and data curation. ZH: writing—original draft preparation. ZH, MK, and NE: writing—review and editing and supervision. MK and NE: visualization. All authors contributed to the article and approved the submitted version.
