# Supplementary figures and images for: The impact of health volunteering of radiology students on improving their self-skills and practical capabilities in the Kingdom of Saudi Arabia
Source: Front Med (Lausanne). 2024 Feb 29;10:1243014. doi: 10.3389/fmed.2023.1243014 (PMC10937525; doi:10.3389/fmed.2023.1243014)

**Supplementary material
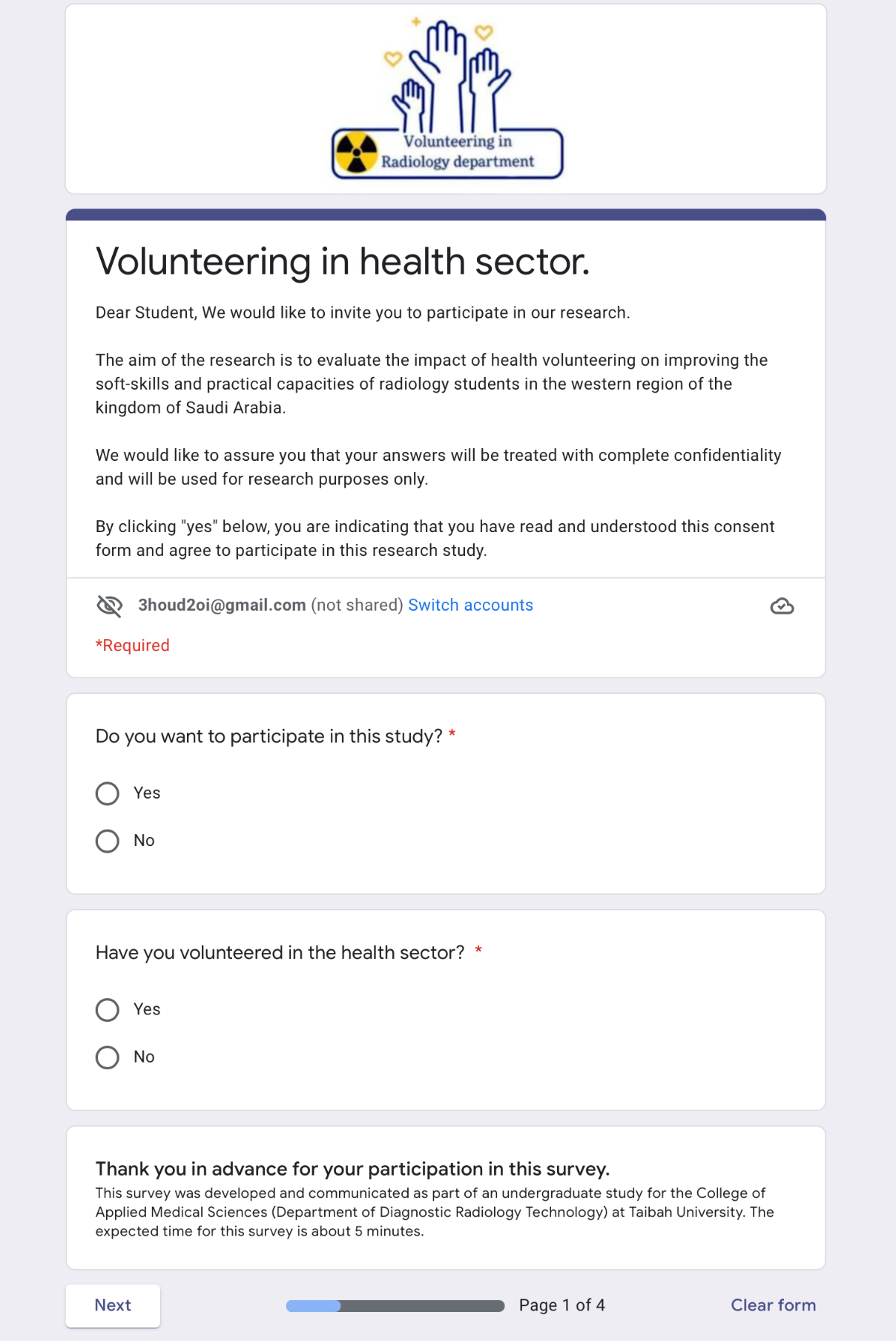
s**

**
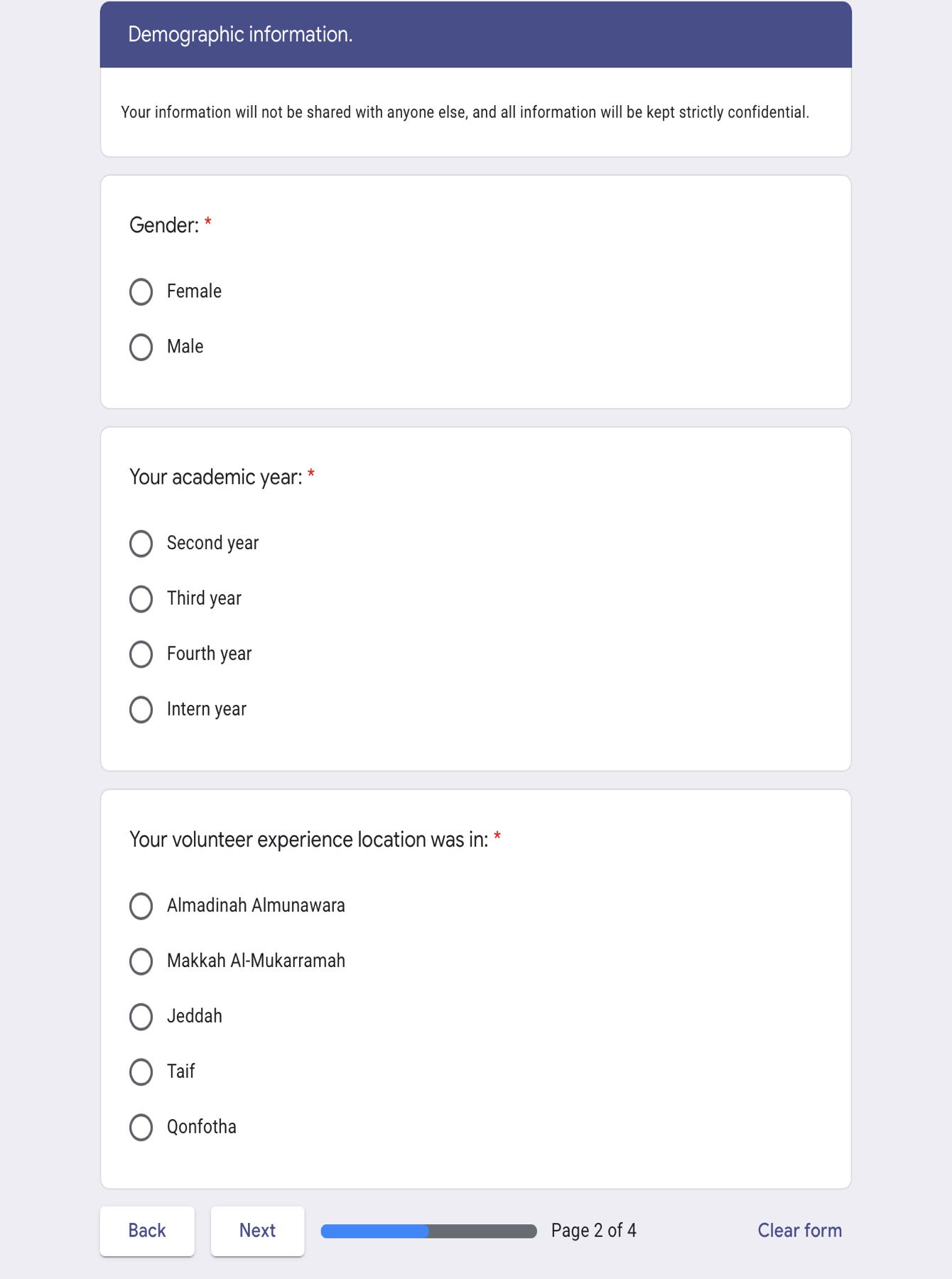
**

**
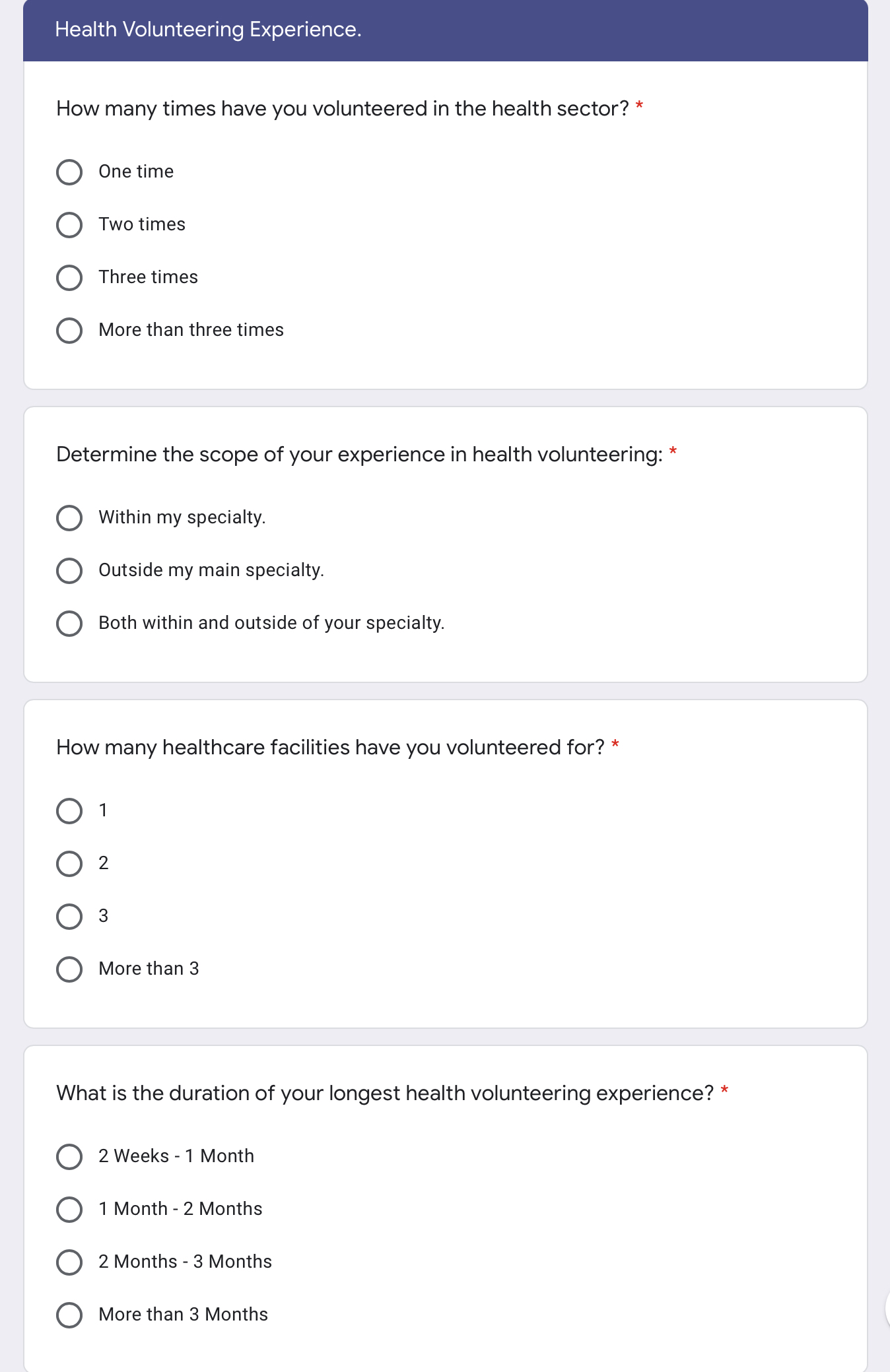
**

**
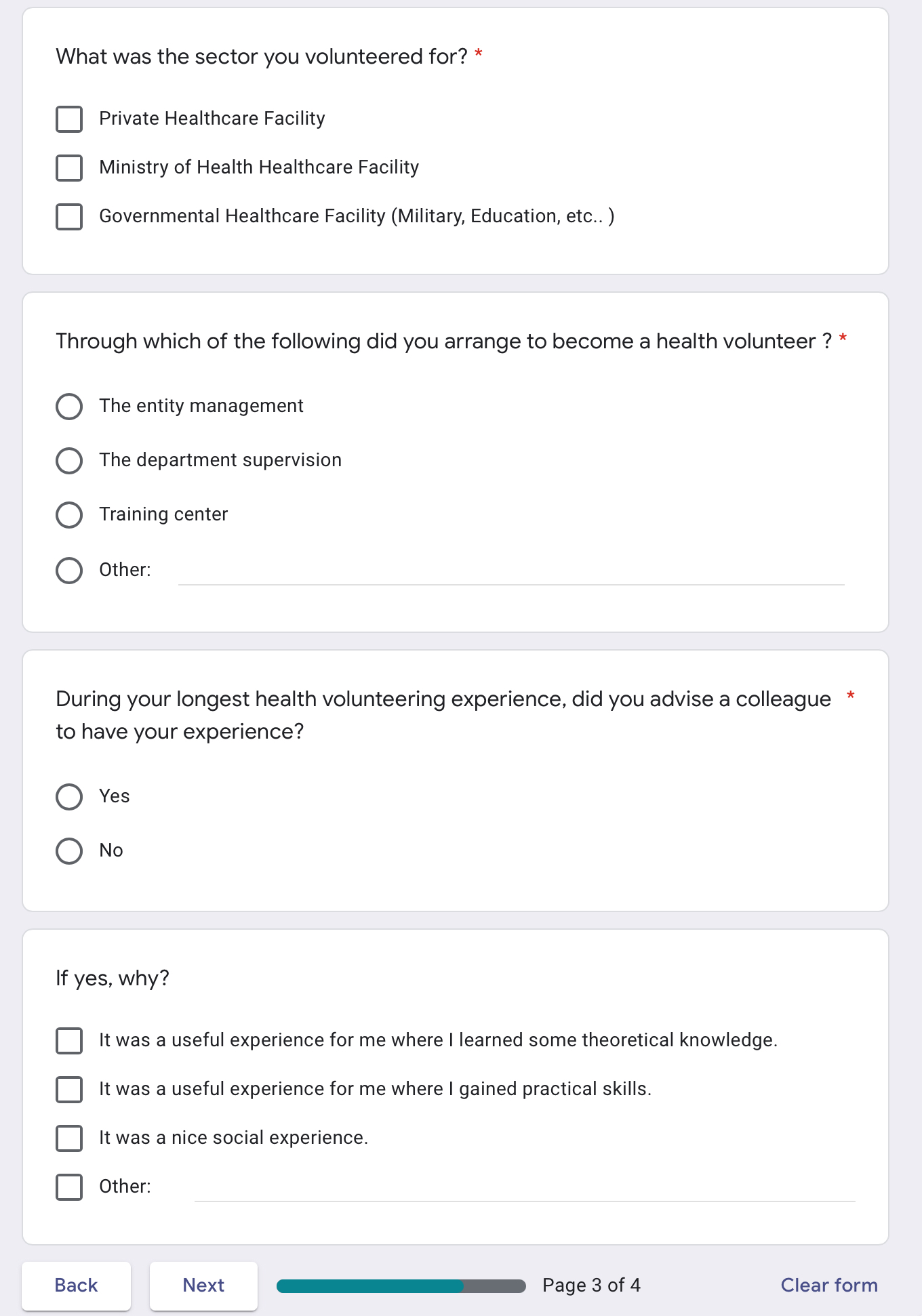
**

**
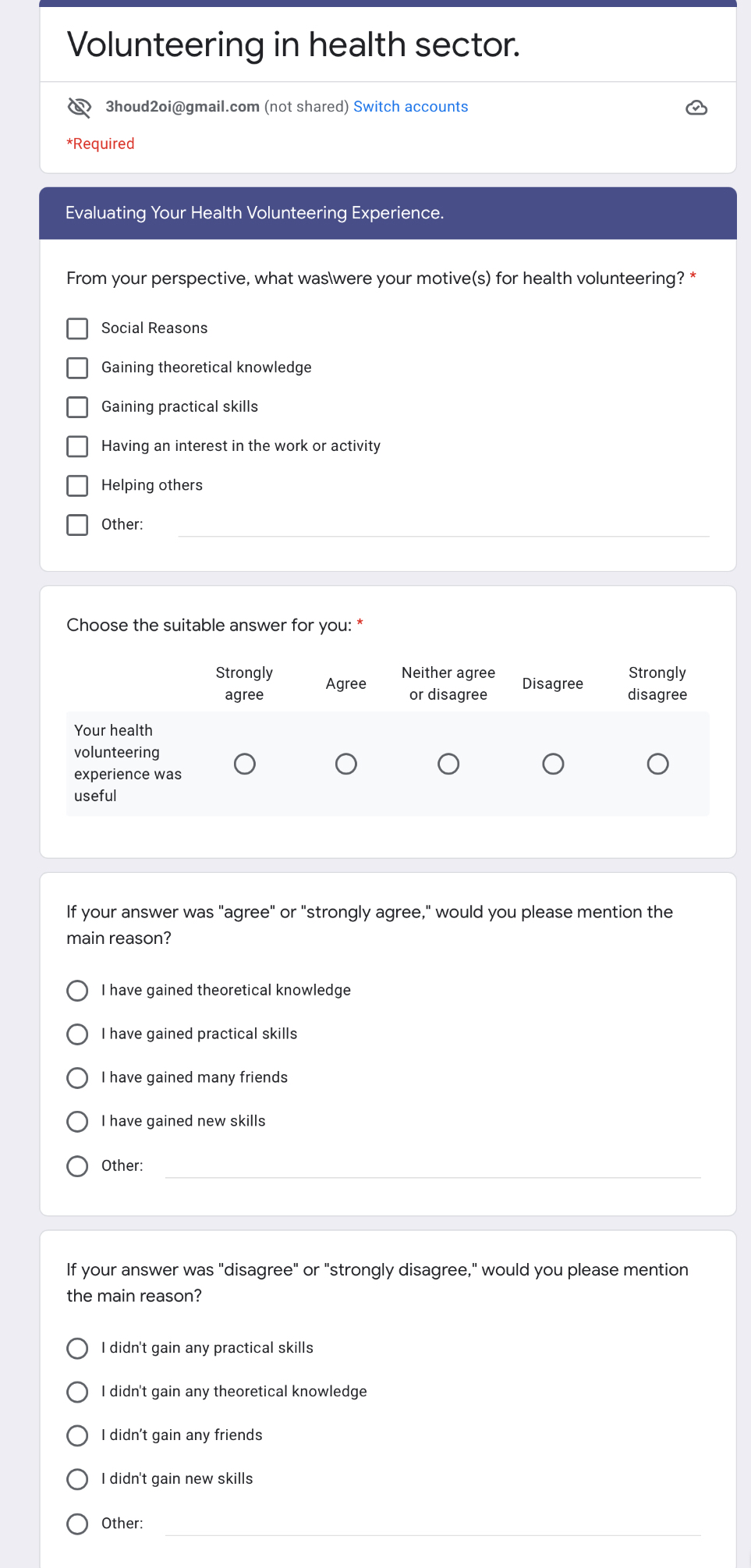
**

**
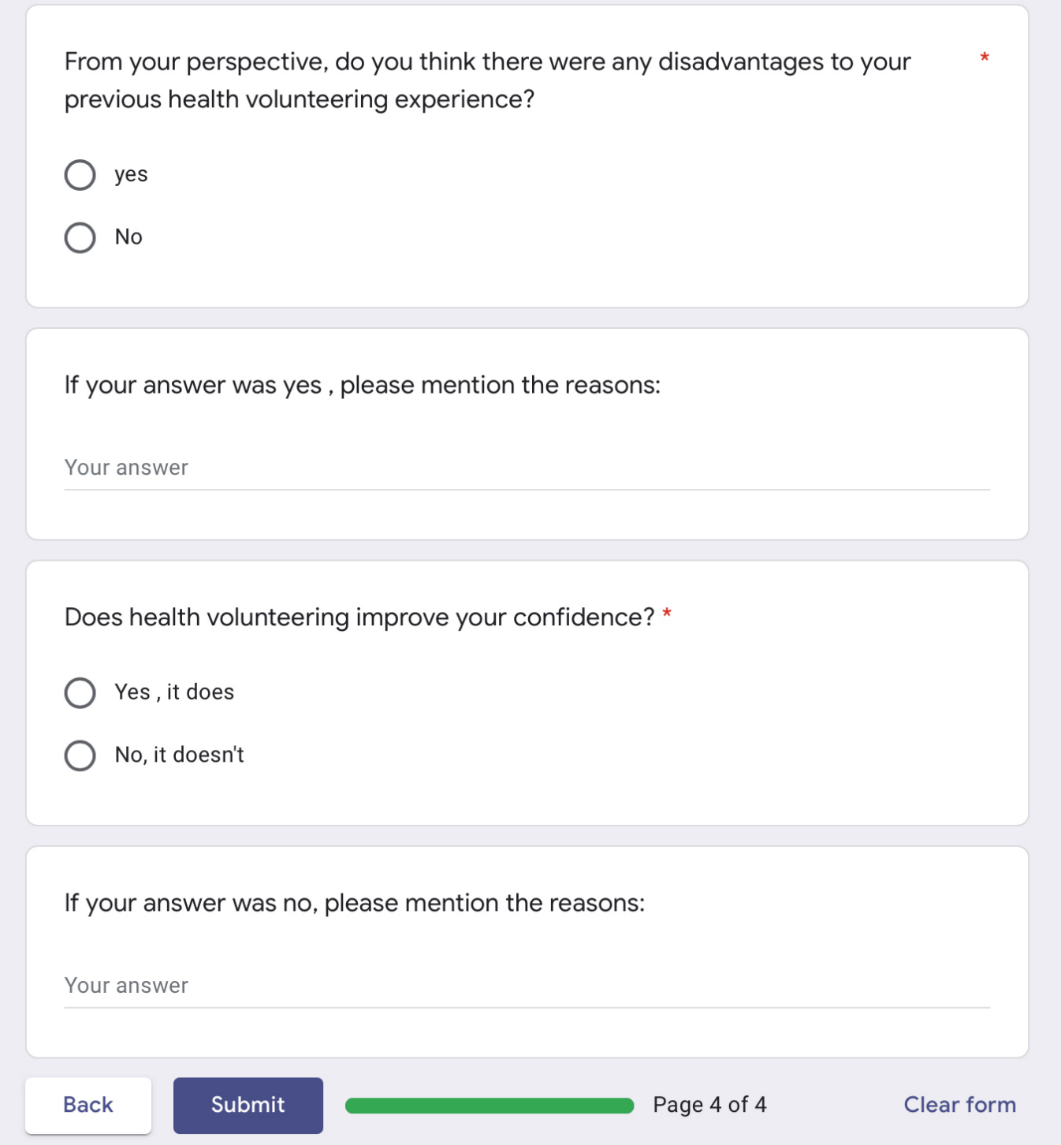

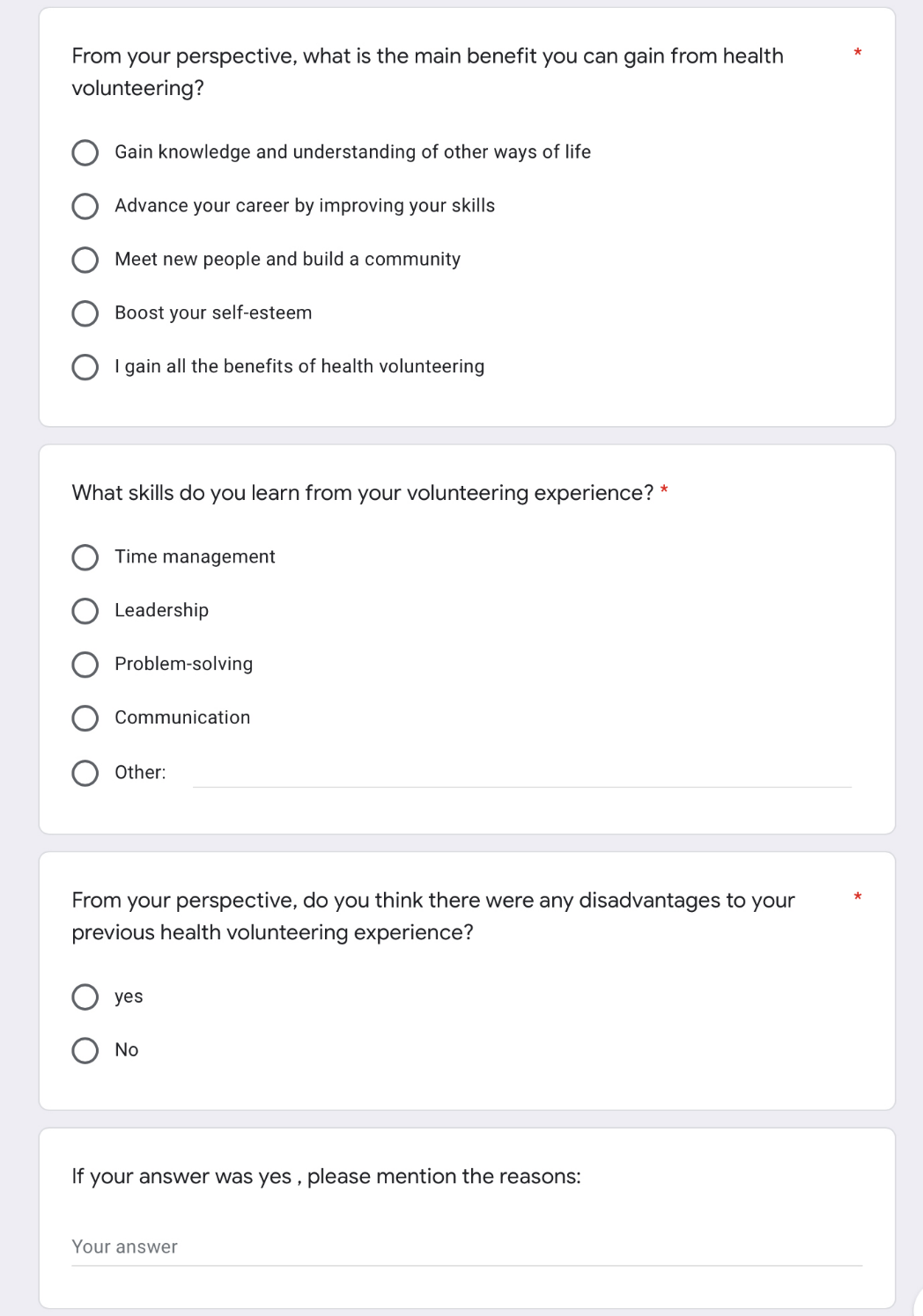
**

Supplement: Supplementary file 1 [file Table_1.DOCX]
